# Micellar Nanocarriers from Dendritic Macromolecules Containing Fluorescent Coumarin Moieties

**DOI:** 10.3390/polym12122872

**Published:** 2020-11-30

**Authors:** Alberto Concellón, María San Anselmo, Silvia Hernández-Ainsa, Pilar Romero, Mercedes Marcos, José Luis Serrano

**Affiliations:** 1 Departamento de Química Orgánica, Instituto de Nanociencia y Materiales de Aragón (INMA), Universidad de Zaragoza-CSIC, 50009 Zaragoza, Spain; aconcell@mit.edu (A.C.); msananselmo@unizar.es (M.S.A.); silviamh83@unizar.es (S.H.-A.); promero@unizar.es (P.R.); m.marcos@csic.es (M.M.); 2 Department of Chemistry, Massachusetts Institute of Technology, Cambridge, MA 02139, USA; 3 ARAID Foundation, Government of Aragón, 50018 Zaragoza, Spain

**Keywords:** dendrimers, liquid crystals, nanocarriers, self-assembly

## Abstract

The design of efficient drug-delivery vehicles remains a big challenge in materials science. Herein, we describe a novel class of amphiphilic hybrid dendrimers that consist of a poly(amidoamine) (PAMAM) dendritic core functionalized with *bis*MPA dendrons bearing cholesterol and coumarin moieties. Their self-assembly behavior both in bulk and in water was investigated. All dendrimers exhibited smectic A or hexagonal columnar liquid crystal organizations, depending on the generation of the dendrimer. In water, these dendrimers self-assembled to form stable spherical micelles that could encapsulate Nile Red, a hydrophobic model compound. The cell viability in vitro of the micelles was studied in HeLa cell line, and proved to be non-toxic up to 72 h of incubation. Therefore, these spherical micelles allow the encapsulation of hydrophobic molecules, and at the same time provided fluorescent traceability due to the presence of coumarin units in their chemical structure, demonstrating the potential of these dendrimers as nanocarriers for drug-delivery applications.

## 1. Introduction

The preparation of polymeric drug nanocarriers is a stimulating topic of research in chemistry, biology and materials science [1,2,3]. In aqueous media, amphiphilic block copolymers self-assemble to minimize energetically unfavorable hydrophobic–water interactions, resulting in a variety of polymeric nanostructures, such as micelles or vesicles [4,5]. Formation of these various morphologies, as well as their size, has been demonstrated to be highly dependent on the composition, molecular geometry, and relative block lengths of the amphiphile. Spherical micelles typically exhibit core-shell architectures in which the hydrophilic shell enables water solubility, whereas the hydrophobic core provides an ideal environment for the encapsulation of hydrophobic molecules. Therefore, polymeric micelles constitute promising instruments to solubilize hydrophobic drugs and release the drug payload at its therapeutic target, while minimizing side effects to healthy tissues [6,7,8].

Dendrimers are ideal candidates for biomedical applications due to their flawless macromolecular structure and exact number of functional groups. For this reason, dendritic amphiphiles have been thoroughly investigated due to their ability to form well-defined nanostructures in water [9,10,11,12,13,14]. Amphiphilic *Janus* dendrimers have demonstrated facile self-assembly in aqueous media to form stable polymeric nanocarriers [15]. These systems are generally prepared by coupling two antagonistic domains, a hydrophilic part and a hydrophobic one. For instance, our group described a series of *Janus* dendrimers that self-assembled in water and could encapsulate several hydrophobic drugs, increasing their water solubility without compromising their therapeutic activity [16,17]. Alternatively, another common approach consists of a hydrophobic dendritic core surrounded by an hydrophilic shell [18]. These amphiphilic dendrimers self-assemble in water generating unimolecular micelles that can load hydrophobic drugs and potentially be internalized into cells due to their small diameters (ca. 5–20 nm).

Although most of these aqueous assemblies were obtained from covalent dendritic systems, it should be considered that the frequently time-consuming synthetic procedures might reduce their practical feasibility in controlled drug release. Therefore, an effective approach to obtain self-assembled systems in water takes advantage of supramolecular ionic interactions to functionalize dendritic cores. In this context, our group reported ionic amphiphilic poly(amidoamine) (PAMAM) dendrimers bearing aliphatic chains that self-assembled in water generating micelles, lamellae or nanospheres [19]. It was also demonstrated that these ionic dendrimers formed complexes with DNA and encapsulated plitidepsin, a hydrophobic drug [20]. However, as drug-delivery systems, all these amphiphilic dendrimers lacked the valuable feature of in vitro traceability.

**Scheme 1:**
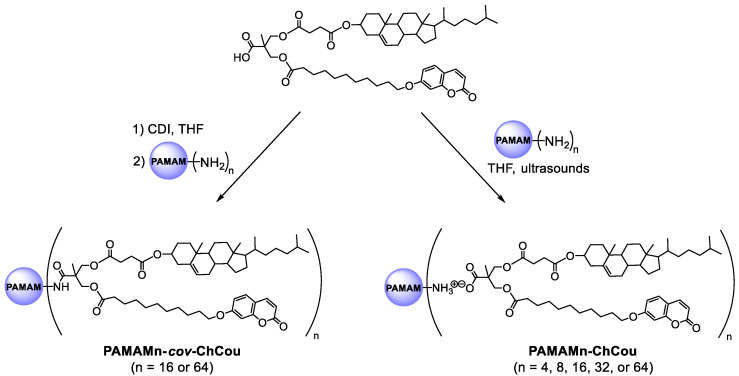
Covalent (**left**) and ionic (**right**) functionalization of PAMAM dendrimer with a hybrid *bis*MPA dendron bearing cholesterol and coumarin moieties.

In the present work, we have prepared polymeric nanocariers that were designed to bear traceable fluorescent groups and to carry a large cargo of hydrophobic drugs. This was accomplished by preparing two families of amphiphilic hybrid dendrimers from hydrophilic PAMAM dendrimer (Scheme 1). PAMAM was surrounded by a hydrophobic *bis*MPA first generation dendron bearing cholesterol (Ch) and coumarin (Cou) moieties. Cholesterol was chosen due to its hydrophobic character as well as its excellent self-assembly properties [12], while coumarin was selected to provide the desired fluorescent traceability. Therefore, **PAMAMn–ChCou** derivatives consist of ionic dendrimers resulting from the supramolecular functionalization of PAMAM generations n = 0 to 4 (bearing 4, 8, 16, 32 or 64 terminal groups), whereas **PAMAMn–*cov*–ChCou** dendrimers come from the covalent functionalization of the second and fourth generation PAMAM dendrimer (bearing 16 or 64 terminal groups, respectively). Herein, we report their synthesis, thermal properties, and morphological aggregation study in water of these amphiphilic dendrimers. Since these dendrimers were designed as polymeric drug carriers, their cytotoxicity in vitro as well as their ability to encapsulate Nile Red as hydrophobic drug model were also studied. Finally, the traceability in vitro was corroborated for both ionic and covalent PAMAM dendrimers.

## 2. Results and Discussion

### 2.1. Synthesis and Characterization of Dendrimers

The preparation of the carboxylic acid dendron (**Ac–ChCou**) was carried out following a previously reported method [21]. Ionic dendrimers (**PAMAMn–ChCou**) were synthesized by mixing a tetrahydrofuran (THF) solution of **Ac–ChCou** with a solution of the corresponding generation of the PAMAM dendrimer in the stoichiometry necessary to functionalize all terminal amine groups (Scheme 1). The mixture was ultrasonicated for 5 min, then THF was slowly evaporated at room temperature, and the sample was dried under vacuum at 40 °C until the weight remained constant. Fourier transform infra-red (FTIR) and nuclear magnetic resonance (NMR) data are consistent with the formation of ionic interactions between PAMAM dendrimers and **Ac–ChCou**.

**Figure 1:**
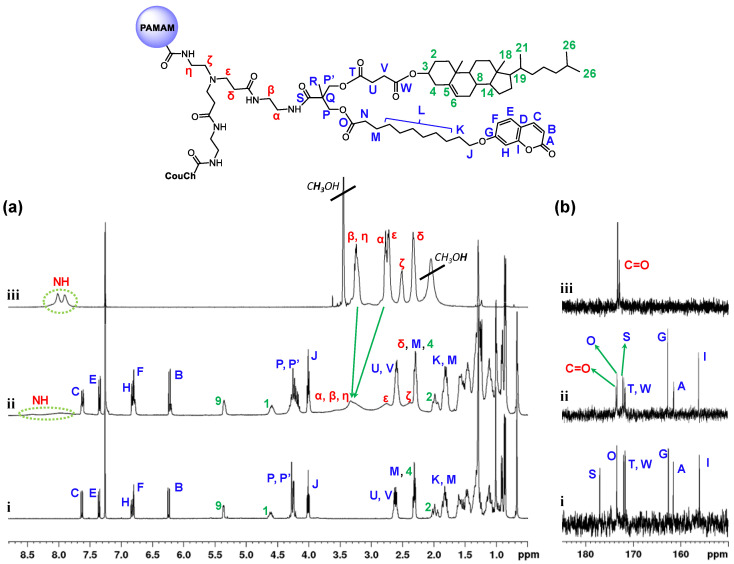
(**a**) ^1^H NMR and (**b**) ^13^C NMR spectra in CDCl_3_ at 25° C of: (i) **Ac–ChCou**, (ii) covalent dendrimer **PAMAM64–*cov*–ChCou** and (iii) **PAMAM64**.

The covalent analogues (**PAMAMn–*cov*–ChCou**) were synthesized by coupling of carbonyldiimidazol (CDI)-activated carboxylic acid group of **Ac–ChCou** with terminal amine groups of the corresponding generation of **PAMAM** (Scheme 1). The FTIR spectra confirmed the formation of the covalent dendrimers. As a representative example, the FTIR spectra of **Ac–ChCou**, **PAMAM16**, and the corresponding covalent dendrimer are shown in Figure S1a in the Supplementary Material. In the spectrum of **PAMAM16–*cov*-ChCou**, the bands at 1730 and 1683 cm^−1^ corresponding to the dimeric form and free form of the carboxylic acid group of **Ac–ChCou** were replaced by a band at 1646 cm^−1^ due to the stretching of the amide carbonyl groups. Further evidence for the formation of the covalent dendrimers was obtained by ^1^H NMR spectra (Figure 1a). The absence of the CH_2_C*H*_2_**–**NH_2_ (*H*_α_, δ = 2.77 ppm) and C*H*_2_CH_2_**–**NH_2_ (*H*_β_, δ = 3.22 ppm) signals and the appearance of the CH_2_C*H*_2_**–**NHCO (*H*_α_, δ = 3.32 ppm) and C*H*_2_CH_2_**–**NHCO (*H*_β_, δ = 3.32 ppm) signals confirmed a quantitative functionalization of terminal amine groups of PAMAM. In the ^13^C NMR spectra, formation of the amide groups was confirmed by the absence of the carboxyl group signal of the acid (*C*_S_, δ = 176.98 ppm) and the appearance of a carbon signal from the amide carbonyl groups (*C*_S_, δ = 172.16 ppm) (Figure 1b).

Complete functionalization of **PAMAMn–*cov*–ChCou** was also confirmed by size-exclusion chromatography (SEC) and MALDI-TOF mass spectrometry (MS). Effective functionalization of the PAMAM dendrimers with **Ac–ChCou** was assessed by SEC traces based on monomodal and narrow molar mass distributions as well as the shift of the molecular weight distribution peak towards lower retention times, which indicated **PAMAMn–*cov*–ChCou** formation (Figure S1b). MALDI-TOF mass spectrum of **PAMAM16–*cov*–ChCou** revealed the presence of an intense peak at 18,167.1 corresponding to the fully functionalized dendrimer (Figure S1c). The rest of the peaks are associated with statistical defects present in original **PAMAM16** dendrimer [22,23]. On the other hand, the high molecular weight of **PAMAM64–*cov*–ChCou** (Exact Mass: 73,815.6) precluded its measurement by MALDI-TOF MS. This behavior has previously been observed in other covalent dendrimers with high molecular weight [24].

### 2.2. Synthesis and Characterization of Dendrimers

The thermal stability and liquid crystalline properties of **Ac–ChCou** and ionic dendrimers (**PAMAMn–ChCou**) were previously reported [21]. For comparative purposes, we include the most relevant data in Table 1. The thermal stability of covalent dendrimers (**PAMAMn–*cov*–ChCou**) was studied by thermogravimetric analysis (TGA). All the samples showed good thermal stability and in all cases the 5% weight loss temperature (*T*_5%_) was detected at temperatures more than 100 °C above the isotropization point (Table 1).

**Figure 2:**
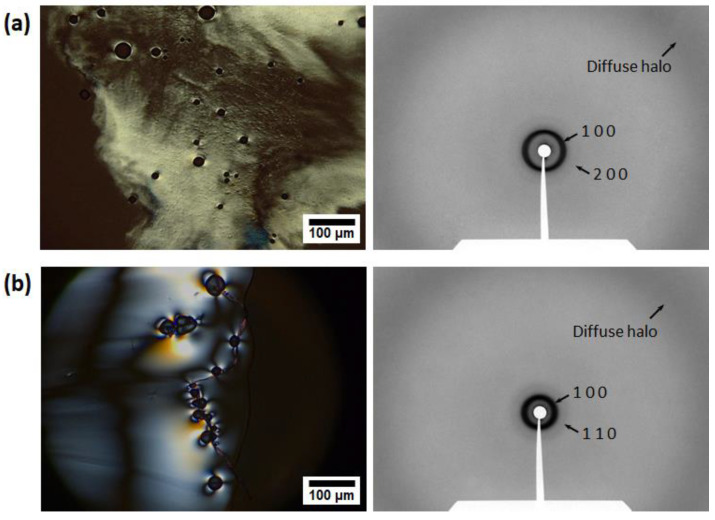
POM microphotographs (**left**) and X-ray diffraction (XRD) patterns (**right**) taken at room temperature for: (**a**) **PAMAM16–*cov*–ChCou** and (**b**) **PAMAM64–*cov*–ChCou**.

Thermal transitions and thermotropic liquid crystal properties were studied by polarized optical microscopy (POM) and differential scanning calorimetry (DSC). The most relevant data are gathered in Table 1. Both **PAMAM16–*cov*–ChCou** and **PAMAM64–*cov*–ChCou** displayed enantiotropic liquid crystal phases, showing birefringent textures by POM (Figure 2). DSC traces showed only a glass transition freezing the mesomorphic order at room temperature. The mesophase-to-isotropic temperatures were taken from POM observations because transition peaks were not detected by DSC.

The nature of the mesophases was identified by X-ray diffraction (XRD). The XRD pattern of **PAMAM16–*cov*–ChCou** contained two sharp maxima in the small-angle region with *d*-spacings in the ratio of 1:2 that were assigned to the first and second order reflections from a lamellar structure. In the high-angle region, a broad diffuse halo was detected at 4.5 Å which is characteristic of the liquid-like order of the hydrocarbon chains (Figure 2a). This kind of XRD pattern is characteristic of a smectic A mesophase due to orthogonal character of the mesophase deduced from the presence of homeotropic domains in POM textures. It has been previously shown that this kind of dendrimers assemble into a smectic A layer, adopting a cylindrical shape, in which the substituents are statistically located upward and downward with respect to the dendrimeric matrix [25]. The XRD pattern obtained for **PAMAM64–*cov*–ChCou** showed two small-angle reflections with *d*-spacings in the ratio of 1:1/√3 arising from the (100) and (110) reflections of a hexagonal columnar lattice (Figure 2b). In the high-angle region, a typical diffuse halo at 4.5 Å, related to the conformationally disordered aliphatic chains, was detected. In this case, the flexibility of the dendrimer matrix allowed a molecular conformation, in which substituents extend radially from the central dendrimer core. The supramolecular organization of these disk-like molecules in columns gives rise to the observed hexagonal columnar mesomorphism [26]. The structural parameters of the covalent dendrimers (**PAMAMn–*cov*–ChCou**) and those of their ionic analogous (**PAMAMn–ChCou**) do not vary to a great extent (Table 1). The slightly higher *d* and *a* values obtained for the covalent dendrimers gives evidence of a higher mobility present in the case of ionic dendrimers where the moieties can fluctuate in position and slightly penetrate into the dendrimer matrix. By contrast, covalent analogues occupy fixed positions.

### 2.3. Self-Assembly of the Dendrimers in Water

**Figure 3:**
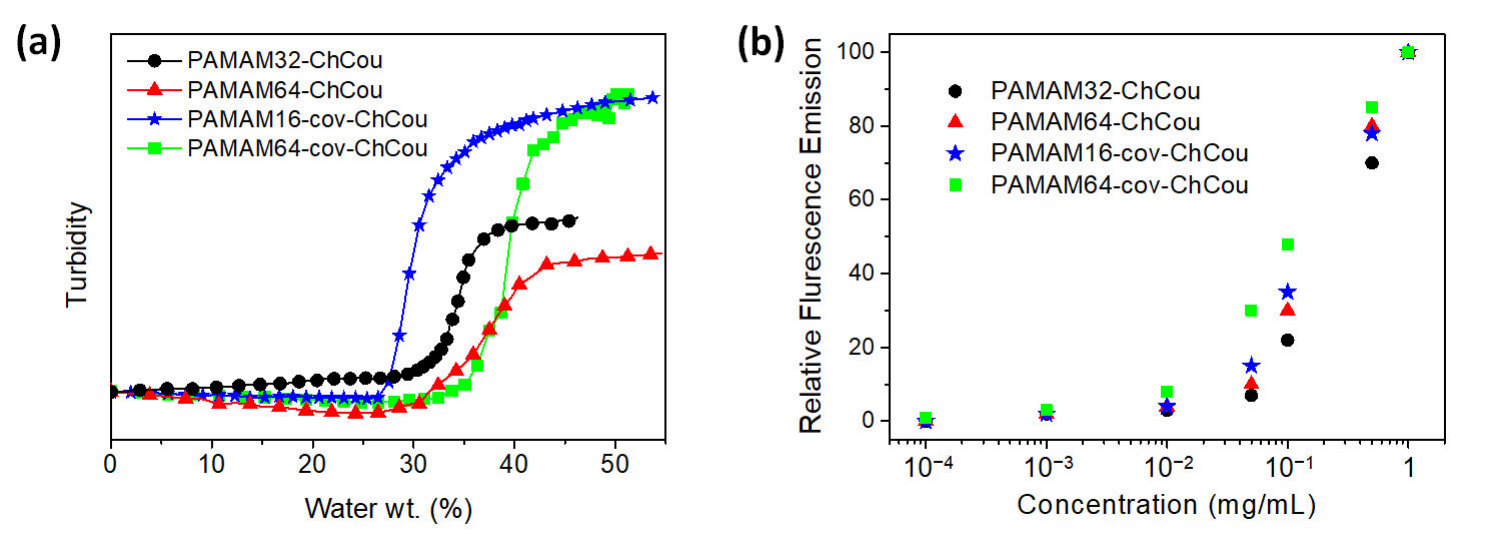
(**a**) Turbidity evolution (THF solutions versus amount of water added). (**b**) Fluorescence intensity of Nile Red at 606 nm (λ_exc_ = 550 nm) versus dendrimer concentration.

Self-assembled nanostructures of the dendrimers were prepared by the co-solvent method using THF/water and following the micellization process by turbidimetry. Dendrimers were first dissolved in THF and then water was slowly added, and at some point, the water addition caused a sudden jump in turbidity which indicated that the dendrimer self-assembly had started (Figure 3a). Once turbidity reached an almost constant value, the resulting dispersion was dialyzed against water to remove the organic solvent. **PAMAM4–ChCou**, **PAMAM8–ChCou**, and **PAMAM16–ChCou** precipitated during dialysis, which pointed to the collapse of the assemblies in water. Nevertheless, stable dispersions were obtained for the rest of the dendrimers after storing for a few weeks. Therefore, any further study was limited to **PAMAM32–ChCou**, **PAMAM64–ChCou, PAMAM16–*cov*-ChCou**, and **PAMAM64–*cov*–ChCou**.

**Figure 4:**
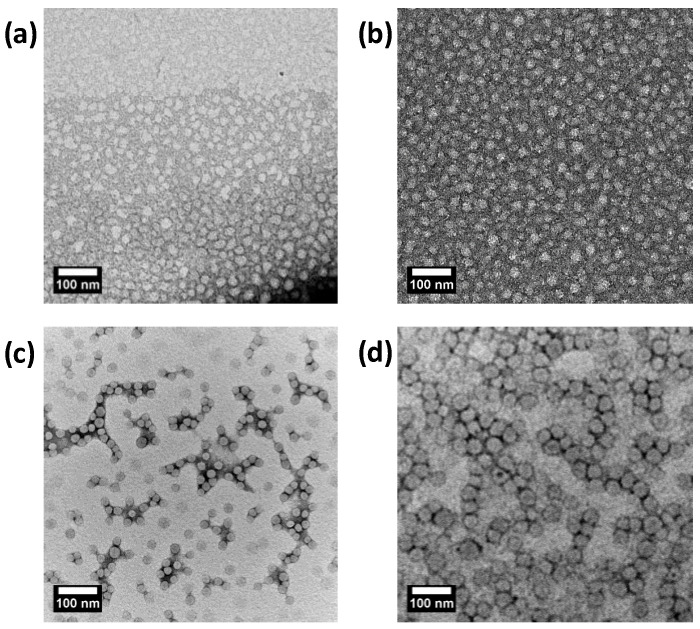
TEM images from water suspensions of: (**a**) **PAMAM32-ChCou**, (**b**) **PAMAM64-ChCou**, (**c**) **PAMAM16-*cov*-ChCou** and (**d**) **PAMAM64-*cov*-ChCou**.

The morphology of the **PAMAM32–ChCou**, **PAMAM64–ChCou, PAMAM16–*cov*–ChCou**, and **PAMAM64–*cov*–ChCou** self-assemblies was first evaluated by transmission electron microscopy (TEM) on dried samples stained by uranyl acetate. TEM images showed the presence of spherical micelles (Figure 4 and Figure S2), whose diameter depended on the dendrimer generation (*D*_h_^TEM^ in Table 2). Because of their amphiphilic nature, dendrimers undergo self-association in water to form spherical micelles in which the hydrophilic PAMAM cores are exposed at the surface, while the hydrophobic parts (coumarin and cholesterol moieties) remain inside the micelle [27]. The average size of the micelles was additionally evaluated by dynamic light scattering (DLS). The mean hydrodynamic diameters (*D*_h_^DLS^ in Table 2) were in good agreement with TEM observations. Two distributions were found in intensity DLS measurements (Figure S2), one corresponding to single micelles and a second distribution, less intense, was attributed to aggregation of single micelles forming a more complex aggregate. However, it should be mentioned that these aggregates were observed in a lower number than single micelles (see the number size distributions obtained by DLS in Figure S2). Number distributions emphasize the species with the highest number of particles (which often tend to be the smaller ones), while intensity distributions emphasize the species with the largest scattering intensity contributing to the overall result (which often tend to be the larger particles) [28,29].

The critical aggregation concentration (CAC) in water, measured by the concentration of the amphiphilic macromolecule above which the macromolecule chains start to associate, was determined by fluorescence using Nile Red as a polarity sensitive probe (Figure 3b) [30,31]. Samples of amphiphilic dendrimers were stirred overnight together with Nile Red at room temperature and the emission spectra of the probe were registered from 560 to 700 nm. The fluorescence spectra showed one emission band at 606 nm which increased on increasing the concentration of the dendrimer. At low concentrations, the weak emission intensities indicated that Nile Red is in water and thus, few micellar self-assemblies are present. At higher concentrations, the emission intensity increases indicating that Nile Red is in a more hydrophobic environment as a result of being encapsulated by the dendrimer self-assemblies. The relationship between fluorescent intensity and the concentration of the dendrimer is non-linear and the onset point corresponds to the CAC. The calculated CAC values are typical for polymeric spherical micelles and are gathered in Table 2.

### 2.4. Absorption and Emission Properties of the Self-Assemblies

**Figure 5:**
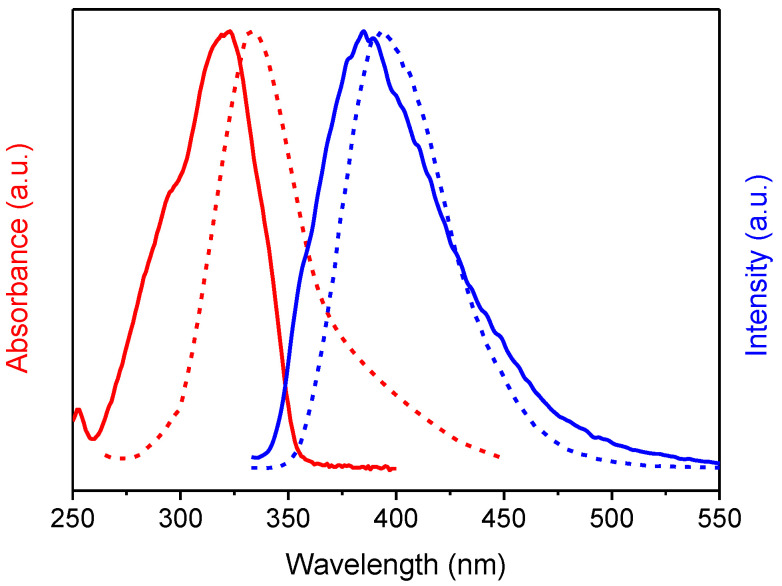
UV-Vis absorption (red) and emission (blue) spectra of **PAMAM64–*cov*–ChCou** self-assemblies in water (dashed lines) and in THF solution (solid lines).

The optical properties of the self-assemblies were evaluated by UV-Vis spectroscopy (Figure 5). The spectra of the amphiphilic dendrimers in THF solution showed the characteristic profile of the coumarin chromophore, which includes an intense band at 323 nm related to the π-π* transition. The UV-Vis absorption spectra of the micelles in water (1 mg/mL) showed broad and bathochromic shifting of the π-π* band in contrast to the results of the dendrimers in THF solution, due to aggregation. The fluorescent traceability of the self-assemblies was evaluated by fluorescence spectroscopy (Figure 5). The emission spectra of the amphiphilic dendrimers in THF exhibited one band at 384 nm, while the spectra of the micelles in water showed a bathochromic shift of around 15 nm, compared to the results in THF solution.

### 2.5. Cytotoxicity and Celullar Uptake of the Self-Assemblies

**Figure 6:**
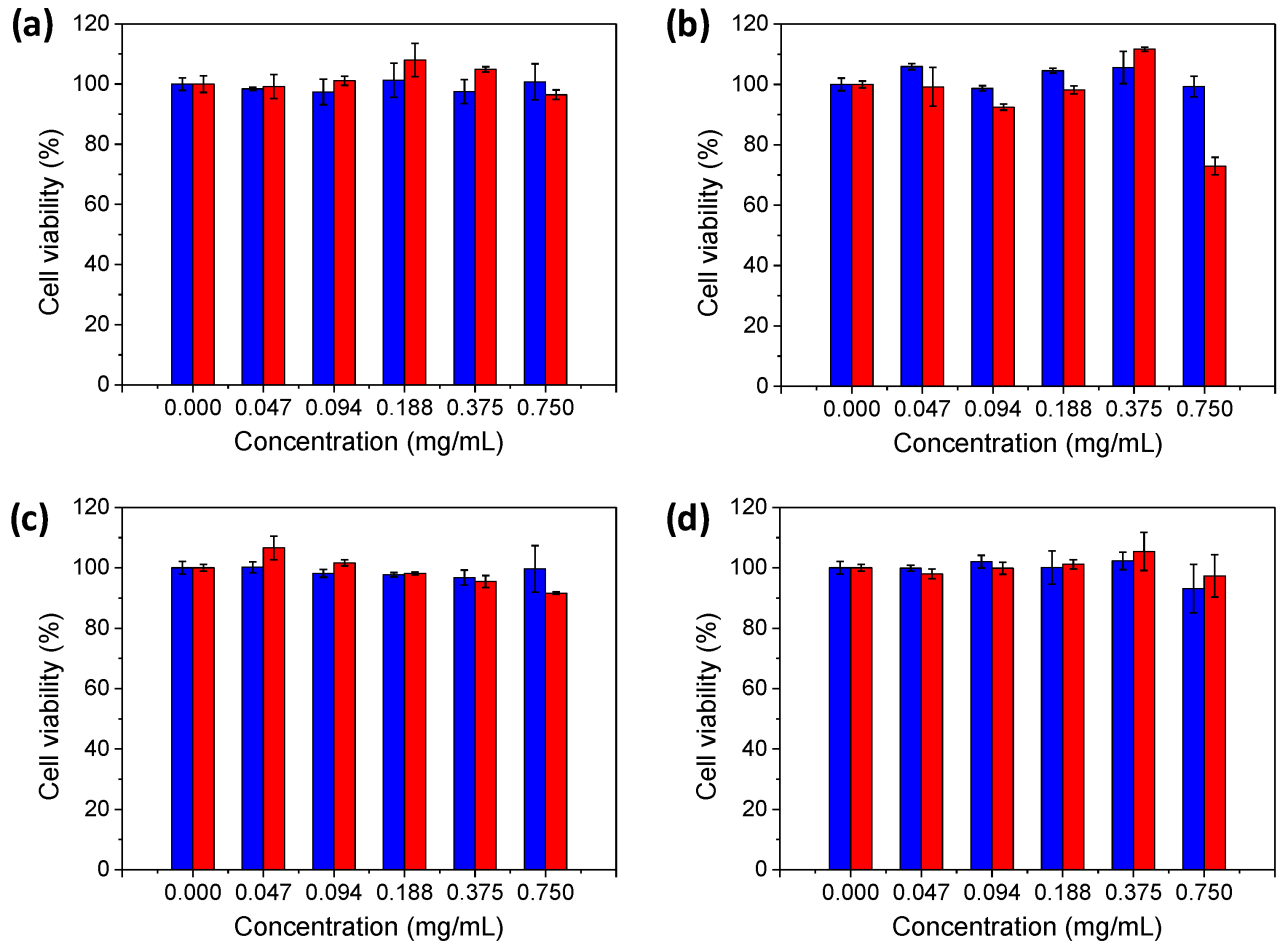
Cell viability in HeLa cell line of: (**a**) **PAMAM32–ChCou**, (**b**) **PAMAM64–ChCou**, (**c**) **PAMAM16–*cov*–ChCou** and (**d**) **PAMAM64–*cov*–ChCou** self-assemblies at increasing concentrations after 24 h (**blue bars**) and 72 h (**red bars**) incubation times.

One essential requirement for these self-assemblies to be used as nanocarriers for biomedical purposes is biocompatibility. To evaluate the cytotoxicity of the micellar self-assemblies, cell viability studies were performed using the HeLa cell line from human cervix cancer. After incubating cells with dendrimers self-assemblies for 24 and 72 h, cell viability was analyzed by the Alamar Blue assay. Briefly, resazurin molecules (blue color) are reduced to resorufin (red color) upon entering living cells. Then, the amount of reduced Alamar Blue active component is directly proportional to the number of metabolically active cells in the culture, which can be spectrophotometrically quantified. In general, high viability values above 90% were obtained for all the dendrimers tested up to 72 h of cell incubation in the presence of increasing concentrations of the self-assemblies from 0.05 to 0.75 mg/mL (Figure 6). However, in the case of **PAMAM64–ChCou**, the ionic assembly of the highest generation, a cytotoxic effect begins to appear after 72 h of incubation at the highest concentration observing a viability decay of up to 70%.

**Figure 7:**
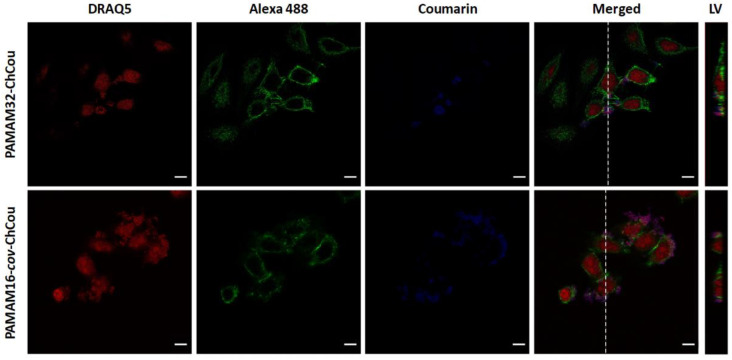
Confocal images of dendrimers **PAMAM32–ChCou** and **PAMAM16–*cov*–ChCou** in HeLa cells after 4 h of incubation at 0.50 mg/mL. Images were acquired at different channels: DRAQ5 (**red**), Alexa 488 (**green**) and coumarin (**blue**). Merged images of these three channels are also shown. LV: Lateral view of the merged images obtained by arranging several confocal Z-stack projections obtained from the points indicated by the white dashed lines. Scale bars: 10 μm.

To evaluate the potential cellular internalization capabilities of ionic and covalent dendrimers, **PAMAM32–ChCou** and **PAMAM16–*cov*–ChCou** were chosen as representative examples of each synthetic strategy because of their similarities in size and their high viability exhibited. Different non-toxic concentrations of the dendrimers, i.e., 0.25, 0.50 and 0.75 mg/mL, were incubated with cells for 4 h at 37 °C and those preparations were later visualized by confocal laser scanning microscopy. Staining of nuclei and actin filaments allowed to recognize cellular morphologies at microscope, and together with the coumarin signal, it was possible to determine the dendrimer location with respect to the cells in culture. It must be noted that in the corresponding controls DRAQ5 seems to be entrapped by dendrimers aside from staining nuclei and thus, coumarin signal turns from blue to purple when channels overlap in the merged images. As no differences were observed among the tested concentrations, images of 0.50 mg/mL of dendrimers are shown as representative examples (Figure 7). An apparent affinity of dendrimers towards HeLa cells was observed since coumarin fluorescence was mostly located in cellular surface. However, no coumarin was detected within the intracellular space as seen in the lateral views of confocal Z-stack projections. A similar behavior was observed with both ionic and covalent structures, thus indicating that the synthetic strategy employed to assemble the dendrons to the dendritic core does not affect in the internalization efficiency in the case of **PAMAM32–ChCou** and **PAMAM16–*cov*–ChCou**.

## 3. Conclusions

We have successfully prepared amphiphilic hybrid dendrimers that consist of a PAMAM dendritic core functionalized with a coumarin-containing *bis*MPA dendron. All the compounds self-assembled in bulk, exhibiting smectic A or hexagonal columnar liquid crystal organizations, depending on the generation of the dendrimer. Moreover, these amphiphilic dendrimers self-assembled in water producing spherical micelles whose size can be modulated by the generation of the dendrimer. Interestingly, these nanocarriers presented the valuable feature of traceability due to the presence of fluorescent coumarin moieties in the chemical structure of the dendrimer. We tested the cell viability of the micelles as a function of their concentration in HeLa cell line, and all the micelles have proven to be non-toxic up to 72 h of incubation below 0.75 mg/mL. Moreover, the traceability of the assemblies thanks to the coumarin moieties has been demonstrated in vitro. We have also demonstrated that these micellar self-assemblies can act as versatile capsules for Nile Red (a model hydrophobic compound). Therefore, we can conclude that these dendrimers may be useful in the preparation of fluorescent polymeric nanocarriers for biomedical applications. Future work will be focused on the study of these amphiphilic dendrimers for effective loading of hydrophobic drugs and their engineering to improve their cellular internalization capabilities.

## Figures and Tables

**Table 1:** Thermal properties and structural parameters.

	T_5%_^a^ (°C)	Phase Transitions ^b^	d_obs_^c^ (Å)	*h k l* ^d^	XRD Parameters
**PAMAM16-*cov*-ChCou**	244	g 25 SmA 71 ^e^ I	46.5	1 0 0	d = 46.8 Å
			23.5	2 0 0	
			4.5 (br)		
**PAMAM16-ChCou** ^f^	209	g 21 SmA 63 ^e^ I	46.2	1 0 0	d = 46.2 Å
			23.1	2 0 0	
			4.5 (br)		
**PAMAM64-*cov*-ChCou**	217	g 35 Col_h_ 87 ^e^ I	53.3	1 0 0	a = 61.5 Å
			30.8	1 1 0	
			4.5 (br)		
**PAMAM64-ChCou** ^f^	198	g 29 Col_h_ 81 ^e^ I	49.3	1 0 0	a = 56.7 Å
			28.2	1 1 0	
			4.5 (br)		

^a^ Temperature (°C) at which 5% mass lost is detected in the thermogravimetric curve. ^b^ differential scanning calorimetry (DSC) data of the second heating process at a rate of 10 °C/min. g: glass, SmA: smectic A mesophase, Col_h_: hexagonal columnar mesophase, I: isotropic liquid. ^c^ *d* value calculated according to Bragg’s equation. ^d^ Miller indices. ^e^ polarized optical microscopy (POM) data. ^f^ Data from Reference [21].

**Table 2:** CAC values and average size diameters (*D*_h_) of the spherical micelles.

	CAC (μg/mL)	*D*_h_^TEM^ (nm) ^a^	*D*_h_^DLS^ (nm) ^b^
**PAMAM32-ChCou**	65	23 ± 4	28 ± 8
**PAMAM64-ChCou**	46	30 ± 5	38 ± 10
**PAMAM16-*cov*-ChCou**	28	18 ± 3	24 ± 6
**PAMAM64-*cov*-ChCou**	19	40 ± 6	47 ± 13

^a^ Average size diameter obtained by TEM from 200 individual micelles. ^b^ Average size diameter obtained by DLS from number size distributions.

## Supplementary Materials

The following are available online at https://www.mdpi.com/2073-4360/12/12/2872/s1. Materials and Characterization Techniques; Experimental Procedures; Supplementary Figures: Figure S1 (FTIR spectra), and Figure S2 (TEM and DLS).
