# Supplementary material for: Micellar Nanocarriers from Dendritic Macromolecules Containing Fluorescent Coumarin Moieties
Source: Polymers (Basel). 2020 Nov 30;12(12):2872. doi: 10.3390/polym12122872 (PMC7761282; doi:10.3390/polym12122872)
Supplement: Supplementary file 1 [file polymers-12-02872-s001.pdf]

Supporting Information

Micellar Nanocarriers from Dendritic Macromolecules Containing Fluorescent Coumarin Moieties

Alberto Concellón[a,‡], María San Anselmo[a], Silvia Hernández-Ainsa[a,b], Pilar Romero[a], Mercedes Marcos[a], and José Luis Serrano[a,]*

[a] Instituto de Nanociencia y Materiales de Aragón (INMA), Departamento de Química Orgánica, Universidad de Zaragoza-CSIC, 50009 Zaragoza, Spain.

[b] ARAID Foundation, Government of Aragon, Zaragoza 50018, Spain.

[‡] Present address: Department of Chemistry, Massachusetts Institute of Technology, Cambridge, MA 02139, USA.

* Correspondence: joseluis@unizar.es

1. MATERIALS AND CHARACTERIZATION TECHNIQUES

Poly(amidoamine) dendrimers (PAMAM) were purchased from Dendritech, Inc. All the reagents were purchased from Sigma-Aldrich and used as received without further purification. Anhydrous THF was purchased from Scharlab and dried using a solvent purification system. The syntesis of the carboxylic acid dendron (**Ac-ChCou**) and the ionic dendrimers **PAMAMn-ChCou** (n= 0–4) was previously described.[1]

Infrared Spectroscopy (IR) spectra were obtained on a Bruker Vertex 70 FT-IR spectrophotometer using KBr pellets.

Nuclear Magnetic Resonance (NMR) experiments were carried out on Bruker Avance spectrometers operating at 400 MHz for ^{1}H and 100 for ^{13}C, using standard pulse sequences. Chemical shifts are given in ppm relative to TMS and the residual solvent peak was used as internal reference. The unequivocal assignment of the signals was verified using 2D NMR experiments: COSY, HSQC and HMBC.

Elemental Analysis was performed using a Perkin-Elmer 2400 microanalyzer.

MALDI-TOF Mass Spectrometry (MS) was performed on an Autoflex mass spectrometer (Bruker Daltonics) using dithranol as matrix.

Size Exclusion Chromatography (SEC) was carried out on a Waters e2695 Alliance liquid chromatography system equipped with a Waters 2424 evaporative light scattering detector using two Styragel® columns, HR4 and HR1 from Waters. Measurements were performed in THF using a flow of 1 mL/min and poly(methyl methacrylate) (PMMA) narrow molar mass standards.

Polarized-Light Optical Microscopy (POM) was performed using an Olympus BH-2 polarizing microscope fitted with a Linkam THMS600 hot stage.

Thermogravimetric Analysis (TGA) was performed using a Q5000IR from TA instruments at a heating rate of 10 ^{o}C/min under nitrogen atmosphere.

Differential Scanning Calorimetry (DSC) was performed using a DSC Q2000 from TA instruments with powdered samples (2–5 mg) sealed in aluminum pans. Glass transition temperatures (T_g) were determined at the half height of the baseline jump, and first order transition temperatures were read at the maximum of the corresponding peak.

X-Ray Diffraction (XRD) was performed with an evacuated Pinhole camera (Anton-Paar) operating a point-focused Ni-filtered Cu-K_α beam. The patterns were collected on flat photographic films perpendicular to the X-ray beam. Powdered samples of the supramolecular complexes were placed in Lindemann glass capillaries (0.9 mm diameter).

[1] A. Concellon, T. Liang, A. P. H. J. Schenning, J. L. Serrano, P. Romero & M. Marcos. *J. Mater. Chem. C* **2018**, *6*, 1000-1007.

Ultraviolet-visible and *Fluorescence* spectra were recorded on an ATI-Unicam UV4-200 spectrophotometer and Perkin-Elmer LS 50B fluorescence spectrophotometer, respectively. All the measurements were performed in dilute THF solutions (10^{-5} to 10^{-7} M) using quartz cuvettes.

Transmission Electron Microscopy (TEM) was performed using JEOL-2000 FXIII electron microscope operating at 200 kV. For the preparation of TEM samples, 5 µL of a 0.5 mg/mL water dispersion of the self-assemblies were deposited onto carbon-coated copper grids, and the water was then removed by capillarity using filter paper. The samples were stained with uranyl acetate and the grid was left to dry overnight under vacuum. The 0.5 mg/mL suspension was prepared by diluting the former suspension with Milli-Q water.

Dynamic Light Scattering (DLS) measurements were carried out in a Malvern Instrument Nano ZS using a He–Ne laser with a 633 nm wavelength, a detector angle of 173° at 25 ^{o}C. The self-assemblies concentrations were 0.10 mg/mL and size measurements were performed at least three times on each sample to ensure reproducibility.

2. EXPERIMENTAL PROCEDURES

2.1 Synthesis of the Covalent and Ionic Dendrimers

The synthesis of the covalent dendrimers (**PAMAMn-*cov*-ChCou**, n= 16 and 64) was performed following a previously described method. Carbonyldiimidazol (CDI) (1.03 eq.) was slowly added to a solution of **Ac-ChCou** (1.00 eq.) in anhydrous THF. The reaction mixture was stirred at RT for 1 h under a stream of argon. The mixture was transferred to a Schlenk flask containing the corresponding PAMAM dendrimer (1.00 eq. terminal amino groups), and it was heated at 40 °C for 72 h. Then the reaction mixture was partially evaporated and carefully precipitated twice using cold methanol. The dendrimer was dried in a vacuum at RT for 48 h.

PAMAM16-*cov*-ChCou. Yield: 55 %. IR (KBr, ν, cm^{-1}): 3316 (N-H), 3085 (=C-H), 2931 (C-H), 1739, 1646 (C=O), 1613 (Ar), 1158 (C-O). ^1H NMR ($CDCl_3$, 400 MHz, δ, ppm): 8.25-7.74 (m, 44H), 7.68-7.58 (m, 16H), 7.39-7.33 (m, 16H), 6.88-6.73 (m, 32H), 6.28-6.17 (m, 16H), 5.45-5.31 (m, 16H), 4.68-4.49 (m, 16H), 4.37-4.08 (m, 64H), 4.05-3.93 (m, 32H), 3.53-3.00 (m, 88H), 2.94-2.20 (m, 264H), 2.04-0.64 (m, 910H). ^{13}C NMR ($CDCl_3$, 100 MHz, δ, ppm): 173.57, 173.36, 172.16, 171.94, 171.74, 162.66, 161.37, 156.14, 143.62, 143.58, 139.77, 128.90, 128.84, 122.87, 113.20, 113.14, 112.58, 101.61, 74.63, 68.86, 56.90, 56.39, 50.25, 46.29, 46.20, 42.51, 39.94, 39.70, 38.25, 37.16, 36.77, 36.39, 35.96, 34.33, 34.26, 32.06, 29.56, 29.46, 29.37, 29.25, 29.17, 29.10, 28.38, 28.17, 27.92, 26.12, 26.07, 25.06, 24.99, 24.46, 24.08, 22.95, 22.71, 21.22, 19.47, 18.90, 17.95, 12.03. MS (MALDI$^+$, dithranol, m/z): calcd. for $C_{1038}H_{1600}N_{58}O_{204}$, 18143.7; found, 18167.1 $[M+Na]^+$. Anal. calcd. for $C_{1038}H_{1600}N_{58}O_{204}$: C, 68.67%; H, 8.88%; N, 4.47%. Found: C, 68.50%; H, 8.65%; N, 5.00%.

PAMAM64-*cov*-ChCou. Yield: 68 %. IR (KBr, ν, cm^{-1}): 3307 (N-H), 3080 (=C-H), 2933 (C-H), 1741, 1641 (C=O), 1613, 1555 (Ar), 1158 (C-O). ^1H NMR ($CDCl_3$, 400 MHz, δ, ppm): 8.67-7.81 (m, 188H), 7.67-7.59 (m, 64H), 7.40-7.32 (m, 64H), 6.88-6.74 (m, 128H), 6.29-6.18 (m, 64H), 5.41-5.26 (m, 64H), 4.70-4.47 (m, 64H), 4.34-4.07 (m, 256H), 4.07-3.90 (m, 128H), 3.52-3.03 (m, 376H), 2.90-2.17 (m, 1128H), 2.05-0.63 (m, 3648H). ^{13}C NMR ($CDCl_3$, 100 MHz, δ, ppm): 173.45, 173.36, 172.14, 172.02, 171.95, 171.65, 171.57, 162.67, 161.46, 156.15, 143.60, 143.51, 139.78, 128.86, 128.83, 122.87, 113.19, 113.15, 113.11, 112.60, 101.62, 74.63, 68.87, 56.91, 56.40, 50.26, 46.46, 46.32, 42.53, 39.95, 39.71, 38.25, 37.17, 36.79, 36.39, 35.96, 34.30, 32.08, 29.59, 29.49, 29.35, 29.28, 29.16, 29.12, 28.38, 28.18, 27.93, 26.12, 26.09, 25.03, 24.46, 24.05, 22.95, 22.71, 21.23, 19.47, 18.90, 18.15, 17.93, 12.03. Anal. calcd. for $C_{4206}H_{6496}N_{250}O_{828}$: C, 68.44%; H, 8.87%; N, 4.74%. Found: C, 68.82%; H, 9.00%; N, 4.62%.

2.2 Preparation of the Self-Assemblies in Water

Milli-Q water was gradually added to a solution of 5 mg/mL of the corresponding dendrimer in THF, and the self-assembly process was followed by measuring the loss of intensity of transmitted light at 650 nm due to scattering (turbidimetry) as a function of the water content. At a critical water value, a sudden increase of turbidity occurs coinciding with the onset of polymer self-assembly. When a constant value of turbidity was reached, the mixture was dialyzed against water to remove THF using a Spectra/Por® dialysis membrane (MWCO 1000) for 4 days. Water suspensions of the self-assemblies were obtained with concentrations around 1.5 mg/mL.

2.3 Determination of the Critical Aggregation Concentration (CAC)

Critical aggregation concentration (CAC) was determined by fluorescence spectroscopy using Nile Red as the probe. 119 μL of a solution of Nile Red in dichloromethane (5×10^{-6} M) was added into a series of flasks and then the solvent was evaporated. Afterwards, water suspensions of the self-assemblies, prepared by diluting the former suspension, were added to each flask with concentrations ranging from 1.0×10^{-4} to 1.0 mg/mL. In each flask a final concentration of 1.0×10^{-6} M of Nile Red was reached and the resulting suspensions were stirred overnight at room temperature to reach equilibrium before fluorescence was measured. The emission spectra of Nile Red were registered from 560 to 700 nm (λ_{exc}= 550 nm).

2.4 Cell Line and Cell Culture

Human cervix cancer cell line HeLa (kindly obtained from Cancer Research UK Cell services) was maintained in DMEM Low-Glucose (with 1 g/L *D*-Glucose, *L*-Glutamine and pyruvate; Gibco) supplemented with 10% FBS and 1% penicillin−streptomycin−amphotericin at 37 ^{o}C and 5% CO_2 in a humidified atmosphere.

2.5 Cytotoxicity Studies: *Alamar Blue Assay*

Cells were seeded at a density of 5 × 10^3 cells per well in 96 multiwell culture plates. After 24 h of incubation, the culture medium was removed, and 100 μL of DMEMc plus the corresponding self-assemblies dispersion in water was added (final dendrimer concentration was 0.05–0.75 mg/mL). Incubation with the self-assemblies was carried out at two different time periods (24 and 72 h). After each incubation time, the solutions were replaced by a 10% v/v *Alamar Blue* dye solution (Thermo Fisher) in fresh DMEMc. Upon entering living cells, resazurin (the active ingredient of *Alamar Blue*, blue in color) is reduced to resorufin (a red compound). After incubation with the reagent for 1.5 h at 37 ^{o}C, changes in viability were detected by absorbance measurement at 570 nm, using 600 nm as a reference wavelength, on a Multiskan GO (ThermoScientific) plate reader. Cells incubated with 100 μL of DMEMc plus the corresponding percentage of milli-Q water (without containing self-assemblies) were used as control. Cytotoxicity is expressed as the relative viability of the cells incubated with the self-assemblies compared to control cells (considered as 100% viability). A total of three replicates per concentration were assayed. The following equation was used to calculate the cell viability:

$$\% = 100 \times \frac{(O_2 \times A_1) - (O_1 \times A_2)}{(O_2 \times P_1) - (O_1 \times P_2)}$$

O_1: molar extinction coefficient of *Alamar Blue* at 570 nm.
O_2: molar extinction coefficient of oxidized *Alamar Blue* at 600 nm.
A_1: Absorbance of test wells at 570 nm.
A_2: Absorbance of test wells at 600 nm.
P_1: Absorbance of positive growth control well at 570 nm.
P_2: Absorbance of positive growth control well at 600 nm.

2.6. *In vitro* cellular distribution of dendrimers

To evaluate the distribution *in vitro* of the fluorescent PAMAM dendrimers previously designed, they were visualized by confocal laser scanning microscopy after incubation with HeLa cells. Briefly, cells were seeded at a density of 4×10^4 cells per well over sterile glass covers on 24 multiwell plates and incubated for 24 h at 37 ºC. Then, medium was replaced with 500 µL of the corresponding dendrimer in complete DMEM at different concentrations, i.e. 0.25, 0.50 and 0.75 mg/mL. After 4 h of incubation at 37 ºC, cells were washed three times with PBS, fixed with 4% (v/v) paraformaldehyde and washed twice with PBS. Cell permeabilization was accomplished with a PBS plus 1% BSA and 0.1% saponin solution and then, actin filaments staining was performed by incubating 1 h in the dark with phalloidin-Alexa Fluor 488 diluted in the permeabilization solution (1:200) Further washings were performed and then, nuclei were stained at the same time as slides mounting with a solution of DRAQ5 in Fluoromount-G mounting medium (1:250). Samples thus prepared were allowed to dry in the dark and sealed for later microscopic observation in a Confocal Zeiss LSM 880 with the 63x oil immersion objective. Coumarin fluorescence was observed at λ_{exc}: 405, λ_{em}:410-510 nm; Alexa Fluor 488 at λ_{exc}: 488, λ_{em}:490-633 nm and finally, DRAQ5 for nuclei detection was examined under λ_{exc}: 633, λ_{em}: 661-759 nm. Zen Blue 2.3 software was employed for image analysis.

3. SUPPLEMENTARY FIGURES

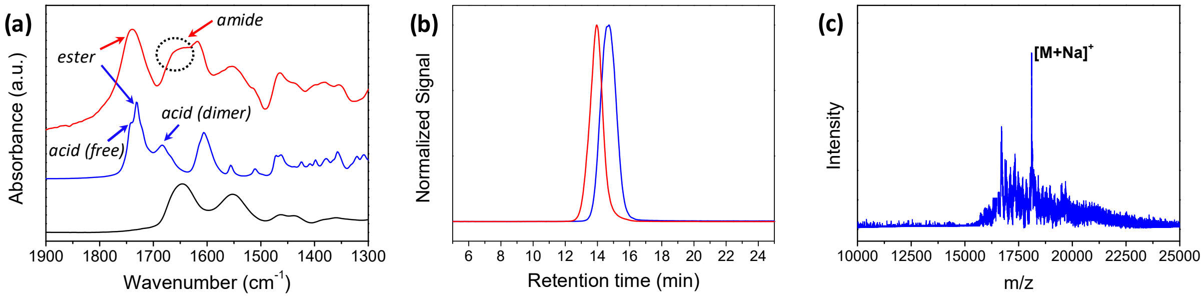

Figure S1. (a) FTIR spectra (C=O *st.* region) of **PAMAM16** (black line), **Ac-ChCou** (blue line) and **PAMAM16-*cov*-ChCou** (red line). (b) SEC traces of: **PAMAM16-*cov*-ChCou** (blue), and **PAMAM64-*cov*-ChCou** (red). **(c)** MALDI-TOF mass spectrum of **PAMAM16-*cov*-ChCou**.

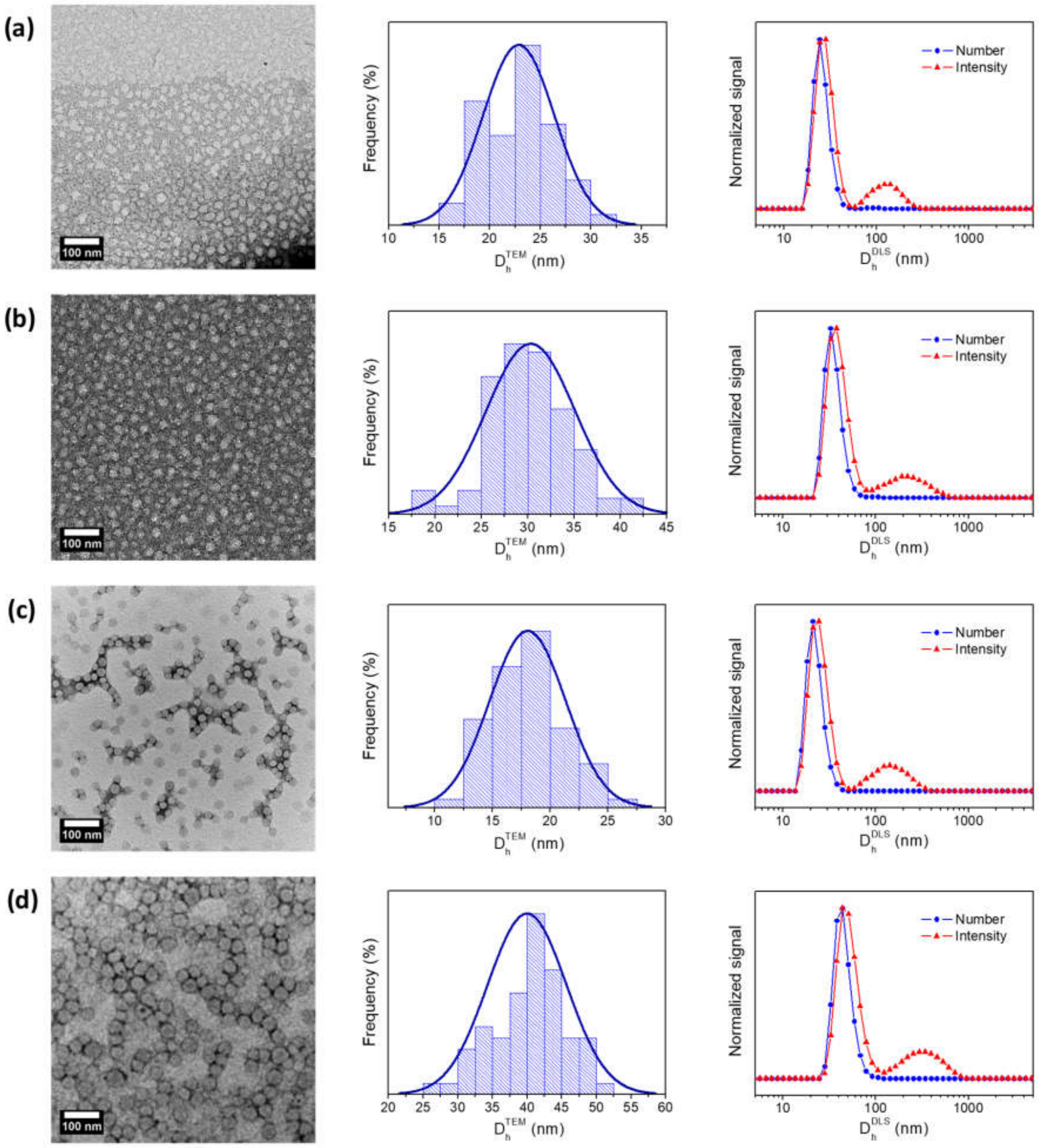

Figure S2. TEM images (left), histogram obtained by TEM from 200 individual micelles (middle), and DLS measurements (number and intensity distributions) (right) from water suspensions of: **(a) PAMAM32-ChCou**, **(b) PAMAM64-ChCou, (c) PAMAM16-*cov*-ChCou** and **(d) PAMAM64-*cov*-ChCou.**
